# Comparative Analysis of Casparian Strip Membrane Domain Protein Family in *Oryza sativa* (L.) and *Arabidopsis thaliana* (L.)

**DOI:** 10.3390/ijms25189858

**Published:** 2024-09-12

**Authors:** Baoping Xue, Zicong Liang, Yue Liu, Dongyang Li, Peng Cao, Chang Liu

**Affiliations:** 1 College of Agronomy, Shenyang Agriculture University, Shenyang 110866, China; xuebaoping@whu.edu.cn (B.X.); jiuweiconghua@163.com (Z.L.); lidongyang0713@163.com (D.L.); 2 Department of Plant Sciences, College of Life Sciences, Wuhan University, Wuhan 430072, China; 3 Institute of Applied Ecology, Chinese Academy of Sciences, Shenyang 110866, China; yueliu@iae.ac.cn; 4 College of Life and Environmental Science, Wenzhou University, Wenzhou 325035, China; caopeng@wzu.edu.cn

**Keywords:** *CASP* gene family, systematic evolution, Casparian strip, expression pattern

## Abstract

The Casparian strip membrane domain proteins (CASPs) are pivotal for the formation of the Casparian strip (CS) in endodermal cells and play a crucial role in a plant’s response to environmental stresses. However, existing research on the *CASP* gene family in rice and *Arabidopsis* lacks a comprehensive bioinformatics analysis and necessitates further exploration. In this study, we identified 41 *OsCASP* and 39 *AtCASP* genes, which were grouped into six distinct subgroups. Collinearity analysis underscored the pivotal roles of WGD and TD events in driving the evolution of CASPs, with WGDs being the dominant force. On the one hand, the analysis of cis-elements indicated that most *OsCASP* and *AtCASP* genes contain MYB binding motifs. On the other hand, RNA-seq revealed that the majority of *OsCASP* and *AtCASP* genes are highly expressed in roots, particularly in endodermal cells, where *OsCASP_like11/9* and *AtCASP_like1/31* demonstrated the most pronounced expression. These results suggest that *OsCASP_like11/9* and *AtCASP_like1/31* might be candidate genes involved in the formation of the endodermis CS. RT-qPCR results demonstrated that *OsCASP_like2/3/13/17/21/30* may be candidate genes for the ion defect process. Collectively, this study offers a theoretical foundation for unraveling the biological functions of *CASP* genes in rice and *Arabidopsis*.

## 1. Introduction

Plant roots exhibit a remarkable selectivity in absorbing water and mineral nutrients from the soil, playing a pivotal role in the growth and development of plants [1]. Nutrient and water uptake by roots primarily occurs via three distinct pathways: the symplastic pathway, the apoplastic pathway and the coupled transcellular pathway [1,2,3]. Notably, the Casparian strip (CS), a structural feature of endodermal and exodermal cells, functions as a crucial barrier in regulating these transport processes [4,5]. Mainly composed of lignin, the CS serves as an apoplastic barrier, effectively preventing the movement of water and mineral nutrients unfettered into and out of the root stele [1,6,7].

It has been reported that the formation and regulatory mechanisms of the CS involve multiple genes. Firstly, CASPs localize to the CS membrane domain, then recruit AtRBOHF, AtESB1, AtPER64 and AtUCC1 enzymes for lignin polymerization [8,9,10,11]. On the other hand, the CIF1/2 (small peptide)-SGN3 (leucine-rich repeat receptor-like kinase SGN3)-SGN1 (receptor-like cytoplasmic kinase (RLCK)) signaling pathway is considered to play a crucial role in maintaining the integrity of the CS [8,12,13,14,15,16,17]. MYB36 encodes a transcription factor that is a major regulator of the CS formation mechanism. It can regulate the expression of *CASP1*, *ESB1*, *PER64* and *UCC1/2* [13,18]. This intricate regulation underscores the sophistication of plant root systems in ensuring efficient and selective nutrient and water uptake, vital for plant growth and survival.

Among them, CASPs are precisely localized at the position where the CS forms in the endodermal cells of *Arabidopsis* roots, participating in the formation of Casparian strips and helping to establish the apoplastic transport barrier in plants [8]. The role of Casparian strips in plant physiology is similar to that of a physiological barrier or valve, finely regulating the process of nutrient and water entry into the vascular bundle. Disruptions in CS formation often lead to abnormal ion absorption from the soil, which can have detrimental effects on plant growth and development. This is evident from the significant alterations in ion concentrations observed in the aerial parts of plants with defective CS formations [4,8]. Notably, the number of *CASP* genes varies among different plants, with 39 members identified in *Arabidopsis* [8], 19 in rice [8], 48 in cotton [19] and 156 in *Pogostemon cablin* [20]. *Arabidopsis* and rice are commonly employed model plants that have unique advantages in terms of genomes, genetic transformation efficiency and transcriptome data resources, making them widely used in gene function research. However, current studies of the *CASP* gene family in rice and *Arabidopsis* lack comprehensive and systematic bioinformatics analysis and require further exploration.

In this study, we identified 41 and 39 *CASP* genes in rice and *Arabidopsis*, respectively, and conducted a systematic bioinformatics analysis encompassing phylogenetic evolution, gene structure, conserved motifs, duplication events and cis-acting elements. Additionally, we analyzed the tissue-specific expression patterns of *AtCASP* and *OsCASP* genes using RNA-seq data, and *OsCASP_like11/19* and *AtCASP_like1/31* might be candidate genes involved in the formation of the endodermis CS. RT-qPCR experiments revealed that *OsCASP* genes may play a pivotal role in ion absorption, with potential implications for enhancing crop yield and quality, thereby contributing to global food security.

## 2. Results

### 2.1. Identification, Gene Location and Physicochemical Properties of Rice and Arabidopsis CASPs

**Figure 1:**
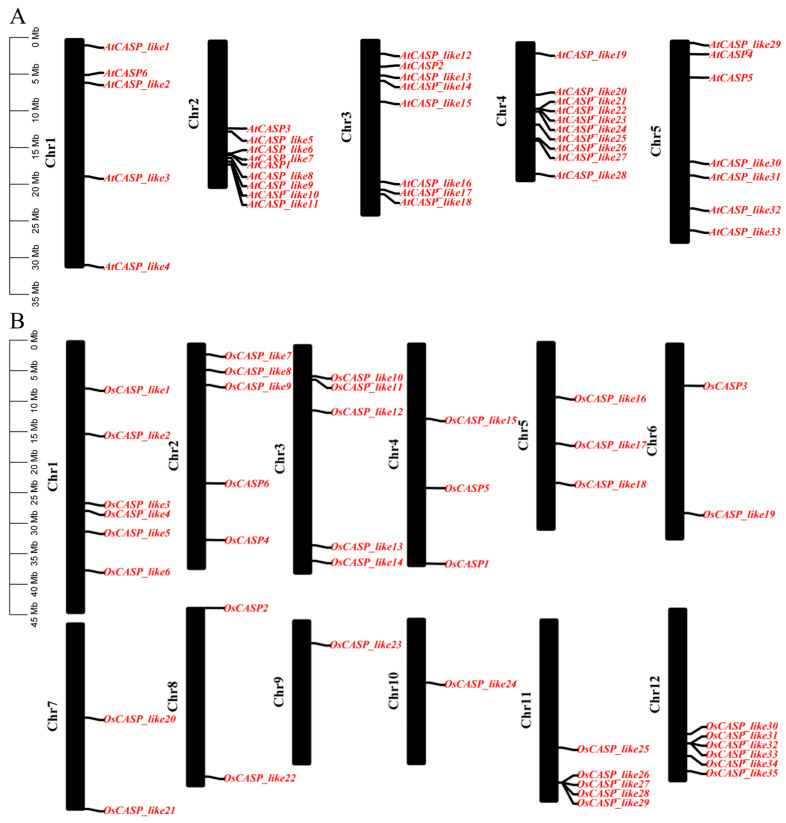
The chromosomal location of the *AtCASP* and *OsCASP* genes in *A. thaliana* and *O. sativa*. (**A**) The chromosomal location of the *AtCASP* genes in *A. thaliana*. (**B**) The chromosomal location of the *OsCASP* genes in *O. sativa*. The chromosomal distribution information of the *CASP* genes was generated from the GFF file information of *A. thaliana* and *O. sativa*.

The hidden Markov model (HMM) of the DUF588 domain (PF04535) was utilized to conduct a thorough search for *CASP* genes in *A. thaliana* and *O. sativa*, resulting in the confirmation of 39 and 41 *CASP* genes, respectively (Supplemental Table S1). Notably, our analysis identified 22 additional CASP members in rice compared to the previously reported 19 members [8], bringing the total number of *CASP* genes in rice to 41. To facilitate identification, we designated these genes as *AtCASP*_*like1*–*AtCASP*_*like33* and *OsCASP*_*like1*–*OsCASP*_*like35*, with the exception of *AtCASP1*–*AtCASP6* and *OsCASP1*–*OsCASP6* (Figure 1), which retained their original nomenclature.

The distribution of *CASP* genes across the chromosomes (Chr) of *A. thaliana* and *O. sativa* was found to be uneven (Figure 1). In *A. thaliana*, Chr 5 and 2 contained the highest number of *CASP* genes, with ten and nine genes, respectively, accounting for 25.64% and 23.08% of the total *CASP* genes (Figure 1A). In rice, Chr 1 and 12 harbored six *CASP* genes each, while Chr 2, 3 and 11 had five CASP genes each. In contrast, Chr 9 and 10 had only a single *CASP* gene (Figure 1B).

Further analysis of the physicochemical properties of the AtCASPs and OsCASPs revealed interesting insights. The number of amino acids in the OsCASPs ranged from 153 (OsCASP_like6) to 421 (OsCASP_like3), while in the AtCASPs, it varied from 152 (AtCASP_like3, AtCASP_like15, AtCASP_like28 and AtCASP_like29) to 297 (AtCASP_like33) (Supplemental Table S1). The molecular weights of the OsCASPs and AtCASPs were found to be between 16,092 Da (OsCASP_like1) and 20,747.65 Da (OsCASP_like35), and 16,092 Da (AtCASP_like1) and 32,611.78 Da (AtCASP_like33), respectively (Supplemental Table S1). The theoretical pI of the OsCASPs and AtCASPs ranged from 4.2 to 10.02, and 4.2 to 10.22, respectively (Supplemental Table S1). Interestingly, we found that the instability index only 5 OsCASPs and 8 AtCASPs proteins are greater than 40, and remaining CASP proteins were less than 40, suggesting that most CASPs in rice and *Arabidopsis* may be stable proteins (Supplemental Table S1). This finding is significant, as stable proteins are more likely to retain their functionality under various cellular conditions. Furthermore, the grand average of hydropathicity of six OsCASPs and three AtCASPs was less than zero; conversely, for thirty-five OsCASPs and thirty-six AtCASPs it was greater than zero (Supplemental Table S1), implying that they are hydrophilic and hydrophobic proteins, respectively.

### 2.2. Phylogenetic Analysis of AtCASPs and OsCASPs

**Figure 2:**
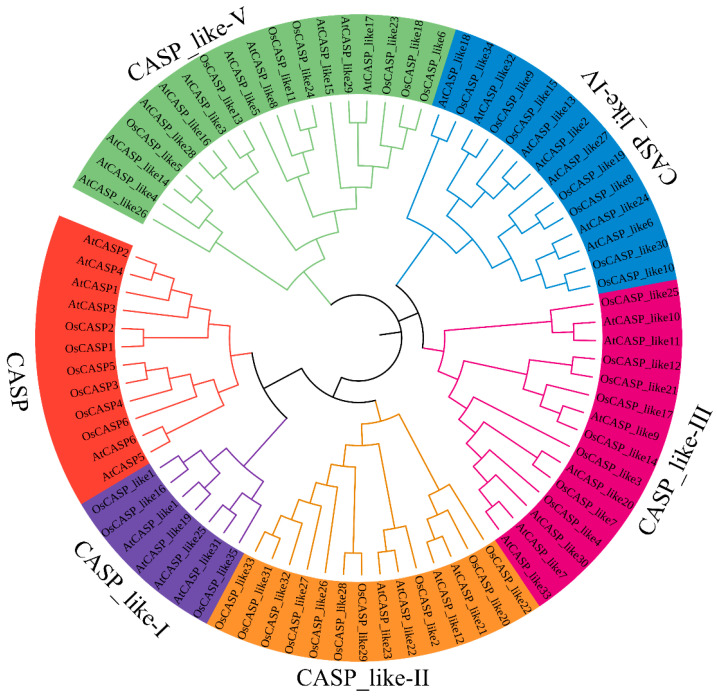
The phylogenetic trees of 39 OsCASPs and 41 AtCASPs. The phylogenetic tree was generated by MEGA 7 (method: Neighbor-Joining; parameter: bootstrap values of 1000 replicates). Red, purple, orange, rose, blue and green represent CASP, CASP_like-I, CASP_like-II, CASP_like-III, CASP_like-IV and CASP_like-V, respectively.

The phylogenetic tree constructed using 39 OsCASPs and 41 AtCASPs provides valuable insights into the evolutionary relationships among these proteins. As shown in Figure 2, these CASPs were divided into six subfamilies (CASP, CASP_like-I, CASP_like-II, CASP_like-III, CASP_like-IV and CASP_like-V) (Figure 2). Furthermore, CASP, CASP_like-I, CASP_like-II, CASP_like-III, CASP_like-IV and CASP_like-V contained twelve (six AtCASPs and six OsCASPs), seven (four AtCASPs and three OsCASPs), fourteen (four AtCASPs and ten OsCASPs), fifteen (seven AtCASPs and eight OsCASPs), fourteen (seven AtCASPs and seven OsCASPs) and eighteen (ten AtCASPs and eight OsCASPs) members, respectively. This variation in subfamily composition could reflect the distinct evolutionary trajectories and functional specializations of CASPs in these two plant species. Previous studies have shown that *AtCASP1*, *AtCASP3* and *OsCASP1* were involved in the formation of the endodermal Casparian strip and the selective uptake of mineral elements [4,8]. Given the close evolutionary ties between CASP and CASP_like-I (Figure 2), it is plausible that the CASP_like-I subfamily genes may also be involved in these processes in rice and *Arabidopsis*. The categories of AtCASPs and OsCASPs confirmed the diversity of their protein structures, and hinted that different subfamily members might have different functions.

### 2.3. Gene Structure and Motif of AtCASPs and OsCASPs

**Figure 3:**
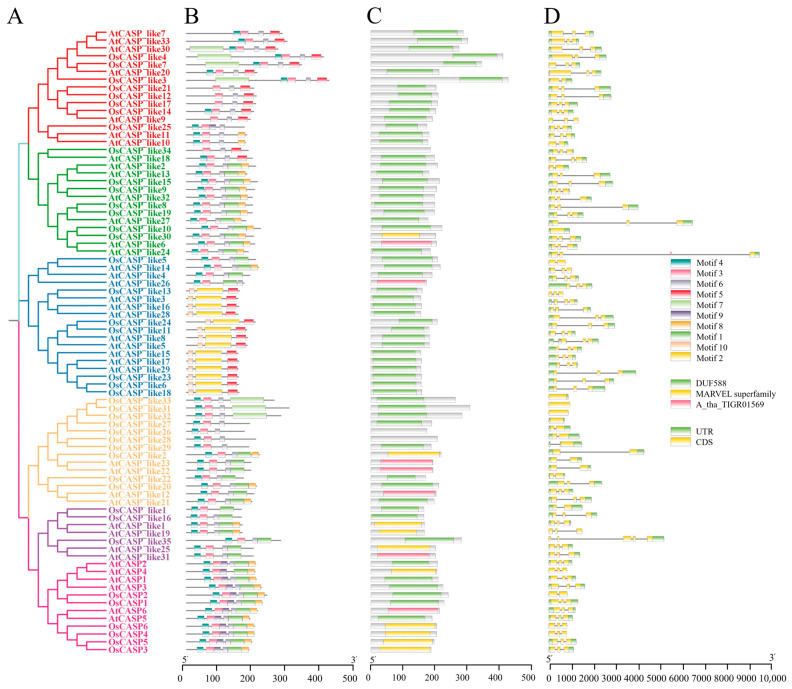
The Phylogenetic tree, conserved motif, conserved domain and gene structure of AtCASP and OsCASP.(**A**) The Phylogenetic tree; (**B**) conserved motif; (**C**) conserved domain; and (**D**) gene structure of AtCASPs and OsCASPs. The different colors in (**A**) represent different subgroups.

The analysis of gene structure and protein conserved motifs provides further insights into the evolution of the *CASP* gene family in *Arabidopsis* and rice. The prevalence of three exons in the majority of *CASP* genes in both species (rice (65.85%) and *Arabidopsis* (89.74%) (Figure 3) suggests a high degree of conservation in the gene architecture of this family. However, the presence of genes with only one or two exons, such as *OsCASP_like27*, *OsCASP_like31*, *OsCASP_like32*, *OsCASP_like33*, AtCASP_like13, *AtCASP_like13*, *AtCASP_like14*, *AtCASP_like22* and AtCASP_like33 (Figure 3), indicates that some members of the family have undergone structural variations during evolution. Moreover, we also analyzed the protein conserved motifs. As shown in Figure 3C, the same subgroups of AtCASPs and OsCASPs had similar conserved motifs. The CASP and CASP_like-I subgroups contained Motif 1, Motif 3, Motif 4, Motif 6, Motif 8 and Motif 9, except for CASP_like1 and CASP_like16 (Figure 3). CASP_like-III contained Motif 2, Motif 5 and Motif 10 (Figure 3). CASP_like-IV contained Motif 1, Motif 3, Motif 4, Motif 6 and Motif 8 (Figure 3). CASP_like-V contained Motif 3 Motif 4, Motif 5, Motif 6, Motif 7, Motif 8 and Motif 9 (Figure 3). Motif 3, Motif 4, Motif 6 and Motif 8 were contained in most CASPs, suggesting that these motifs play important functional roles that are conserved across subgroups. In contrast, the unique motifs present in CASP_like-III (Motif 2 and Motif 10) and the absence of Motif 7 in *Arabidopsis* CASPs suggest that these subgroups have evolved specialized functions. The presence of Motif 7 exclusively in the rice CASPs is particularly noteworthy (Figure 3). This motif may contribute to functional differences between rice and *Arabidopsis* CASPs, highlighting the functional divergence of the CASP gene family during evolution. These results suggest that the CASP genes of *Arabidopsis* and rice have functional divergence and functional conservation during evolution.

### 2.4. Duplication Events, Synteny and Ka/Ks Analysis of AtCASP and OsCASP Genes

**Figure 4:**
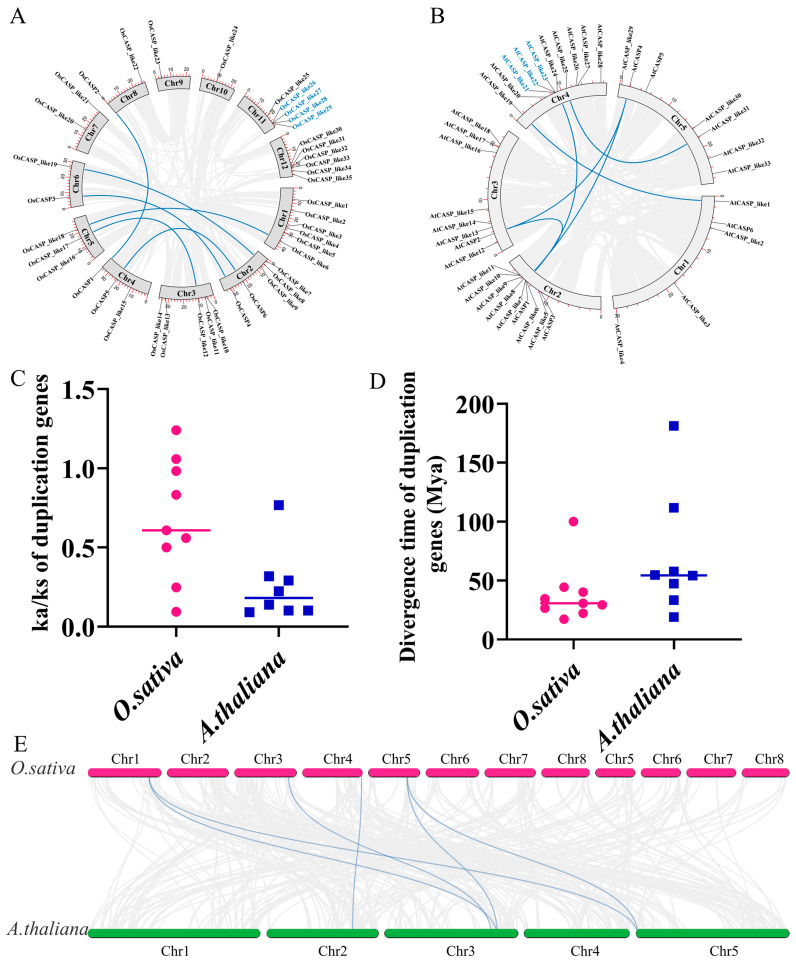
The duplication events, synteny and Ka/Ks analysis of the *CASP* genes in rice and *Arabidopsis*. (**A**) The duplication events of the *CASP* genes in rice. The genes in a bule color represent the tandem duplications and the red lines represent the whole segmental duplication genes. (**B**) The duplication events of the *CASP* genes in *Arabidopsis*. The genes in a blue color represent the tandem duplications and the red lines represent the whole segmental duplication genes. (**C**) The Ka/Ks ratio calculations of the *CASP* gene pairs in rice and *Arabidopsis*. The pink and purple lines represent the median of ka/ks ratio. (**D**) The divergence time predictions of the CASP gene pairs in rice and *Arabidopsis*. The pink and purple lines represent the median of Mya. (**E**) The synteny analysis of the *CASP* genes between rice and *Arabidopsis*. The collinear blocks between rice and *Arabidopsis* are shown by gray lines. The syntenic *CASP* gene pairs between rice and *Arabidopsis* are highlighted by blue lines.

Previous studies have demonstrated that tandem duplications (TDs) and whole genome duplications (WGDs) play pivotal roles in the expansion of the gene families in rice and *Arabidopsis* [21]. To elucidate the expansion mechanisms underlying the expansion of the CASP gene family in these two species, the duplication events of the *AtCASP* and *OsCASP* genes were analyzed. As depicted in Figure 4A,B, our findings revealed a total of twelve WGD gene pairs and five TD gene pairs in rice and *Arabidopsis*. Specifically, we identified three TD gene pairs in rice and two in *Arabidopsis*, whereas six WGD gene pairs were confirmed in both species (Figure 4A,B). These findings highlight the concerted contribution of TDs and WGDs in the generation of the *CASP* gene family in rice and *Arabidopsis*, with WGDs assuming a more prominent role.

To further interrogate whether these homologous *CASP* gene pairs were subjected to selective pressures (including purifying and positive selection), we calculated the Ka (nonsynonymous) to Ks (synonymous) substitution ratio using Tbtools v2.121 (Beijing, China) [22]. Notably, we observed that the Ks/Ka ratios for most homologous gene pairs of *AtCASP* and *OsCASP* were less than one, with the exception of *OsCASP_like26/OsCASP_like27* and *OsCASP_like28/OsCASP_like29*, indicating that these homologous gene pairs were under purifying selection (Figure 4C and Supplemental Table S2). Moreover, the divergence time of most homologous gene pairs ranged from 17.28 to 57.81, except *AtCASP1/AtCASP2*, *AtCASP1/AtCASP4* and *OsCASP4/OsCASP3* (Figure 4D and Supplemental Table S2).

To deeply explore the evolution mechanisms of the *CASPs*, we analyzed the collinearity of the *CASP* genes between rice and *Arabidopsis*. A total of six collinear *CASP* homologous gene pairs of rice and *Arabidopsis* were found. Interestingly, between *Arabidopsis* and rice, some *CASP* genes were identified to be associated with at least two genes, such as *OsCASP_like18/AtCASP_like17* and *OsCASP_like18/AtCASP_like29* (Figure 4E and Supplemental Table S3).

### 2.5. Cis-Element Analysis of AtCASPs and OsCASPs

**Figure 5:**
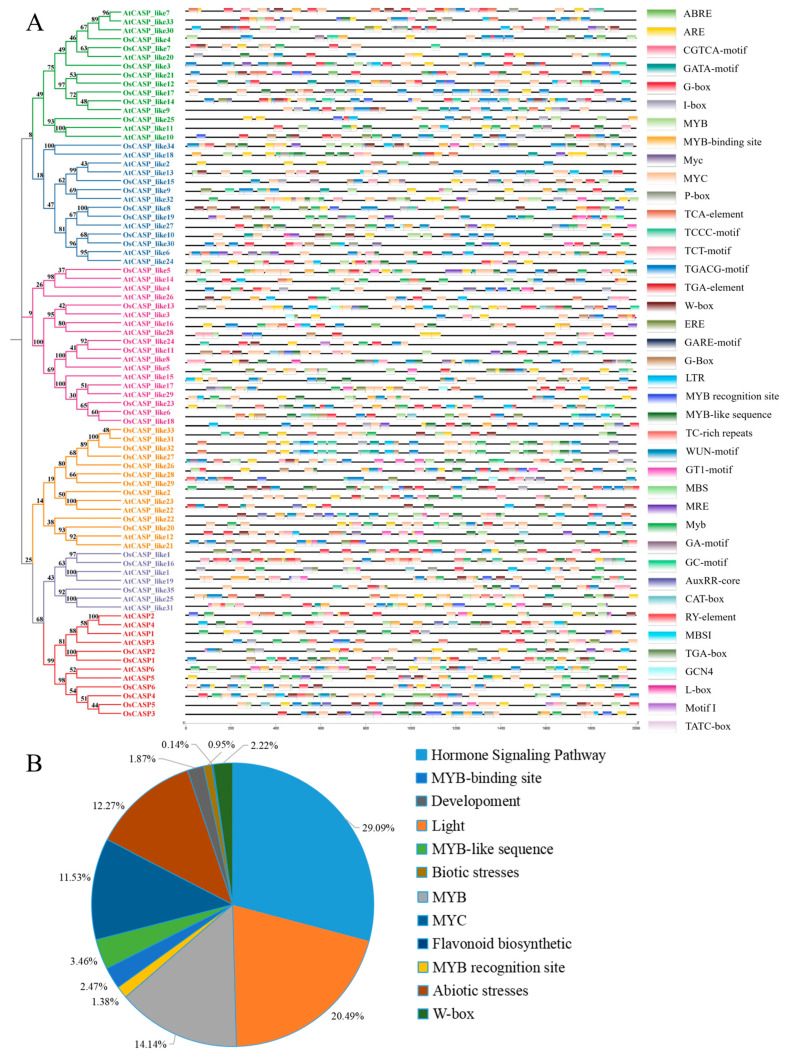
The cis-elements were predicted in 2000 bp promoter sequences of *AtCASPs* and *OsCASPs*. (**A**) The cis-elements were analyzed by plantcare. The different colors represent different subgroups. (**B**) The percentages of the different cis-elements out of all cis-elements.

To delve deeper into the potential functions and regulatory mechanisms of the *CASP* genes in rice and *Arabidopsis*, we analyzed the cis-elements within the promoter regions of the *AtCASP* and *OsCASP* genes using PlantCARE. There are a total of 2810 cis-elements within the 2k promoter sequences of these genes (Figure 5 and Supplemental Table S4). As depicted in Figure 5 and Supplemental Table S4, these cis-acting elements encompass a diverse array of categories, including those related to hormone responses, light perception, abiotic and biotic stress responses, growth and development, and flavonoid biosynthesis. We also found that some WRKY and MYB transcription factors specifically bind to the target gene promoter regions of the cis-acting elements TTGAC (C/T) (W-box) and CAACCA (MYB). Hormone-responsive elements included auxin (AuxRR-core, TGA-element and TGA-box)-, abscisic acid (ABRE)-, salicylic acid (TCA-element)-, gibberellin (GARE-motif, P-box and TATC-box)-, ethylene (ERE)- and methyl jasmonate (CGTCA-motif and TGACG-motif)-responsive elements (Figure 5 and Supplemental Table S4). Interestingly, some *CASP* genes contain multiple hormone-responsive elements. For example, *OsCASP_like1*, *OsCASP_like14*, *AtCASP_like3* and *AtCASP_like10* contain abscisic acid-, salicylic acid-, methyl jasmonate- and ethylene-responsive elements; *OsCASP3*, *OsCASP_like22*, *AtCASP_like5*, *AtCASP_like7*, *AtCASP_like15* and *AtCASP_like33* contain gibberellin- and auxin-responsive elements; *OsCASP4*, *OsCASP_like4*, *OsCASP_like6*, *OsCASP_like12, OsCASP_like13*, *OsCASP_like15*, *OsCASP_like24* and *AtCASP_like9* contain abscisic acid and methyl jasmonate; *OsCASP_like18* contain auxin and methyl jasmonate (Figure 5 and Supplemental Table S4). This result suggests that these genes may be synergistically involved in multiple hormone signaling pathways. In addition, most *AtCASP* and *OsCASP* members contain W-box, MYB and MYC binding sites, which are general stress-responsive elements (Figure 5 and Supplemental Table S4). These findings suggest that CASP genes may play crucial roles in mediating plant responses to various environmental stresses. Additionally, light-responsive elements were identified, implying potential roles in photomorphogenesis and light signaling pathways.

### 2.6. Tissue-Specific Expression Patterns of AtCASP and OsCASP Genes by RNA-Seq

**Figure 6:**
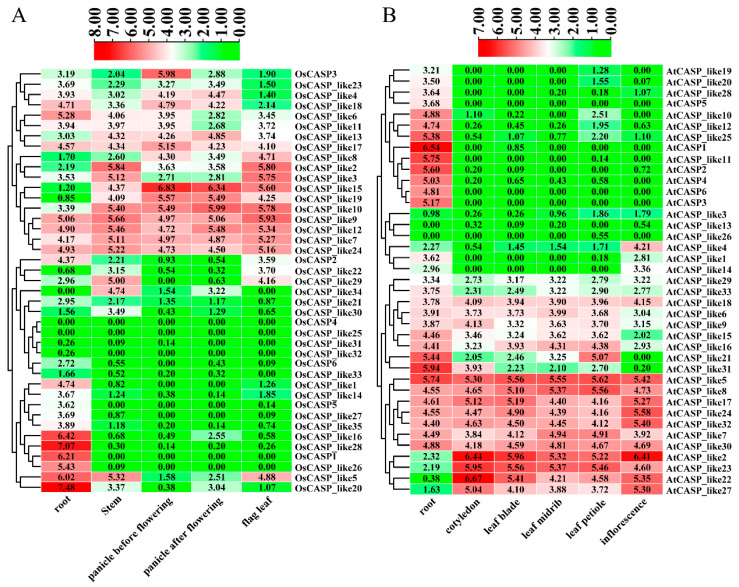
The expression pattern analysis of *CASP* genes in different tissues of rice and *Arabidopsis*. (**A**) The expression patterns of *OsCASP* genes in different tissues (root, stem, panicle before flowering, panicle after flowering and flag leaf). (**B**) The expression patterns of *AtCASP* genes in different tissues (root, cotyledon, leaf blade, leaf midrib, leaf petiole and inflorescence). The heatmap was constructed by Tbtools v2.121 software (Beijing, China). The red and green boxes indicate high and low expression levels of the *AtCASP* and *OsCASP* genes, respectively.

The analysis of transcriptome data across various organs in rice (root, stem, panicle before flowering, panicle after flowering and flag leaf) and *Arabidopsis* (root, cotyledon, leaf blade, leaf midrib, leaf petiole and inflorescence) provided valuable insights into the expression patterns of the *AtCASP* and *OsCASP* genes. Notably, with the exception of a few genes such as *OsCASP4*, *OsCASP_like25*, *AtCASP_like13* and *AtCASP_like26*, the majority of the *CASP* genes exhibit higher expression levels in roots compared to other tissues (Figure 6 and Supplemental Table S5). As shown in Figure 6, approximately half of the *AtCASP* and *OsCASP* genes are widely expressed in different tissues, whereas some genes are highly expressed in specific tissues (Figure 6). For example, *OsCASP1/5/6*, *OsCASP_like1/5/14/16/20/26/27/28/33/35*, *AtCASP1_like10/11/12/19/20/25/28* and *AtCASP1/2/3/4/5/6* (Figure 6 and Supplemental Table S4) are specifically highly expressed in roots. This preferential expression in roots suggests a potentially important role for these genes in root development and function.

**Figure 7:**
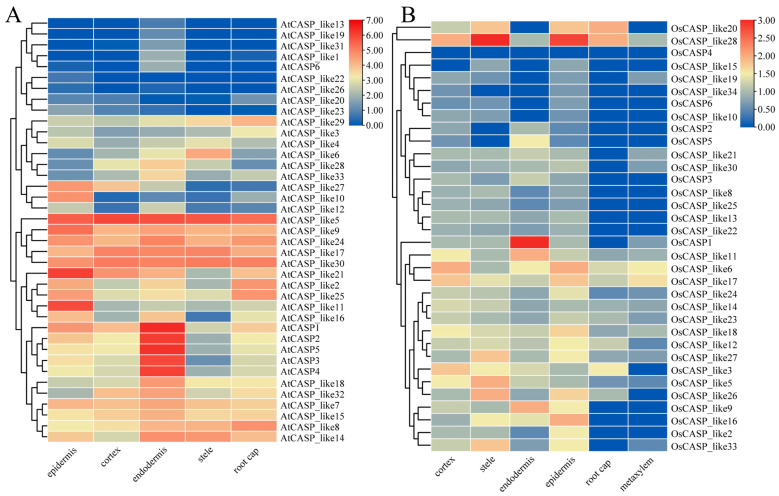
The expression patterns of *CASP* genes in different root cells of *Arabidopsis* and rice. (**A**) The expression patterns of *AtCASP* genes in different root cells (epidermis, cortex, endodermis, stem and root cap). The single-cell transcriptome data of the *AtCASP* genes in *Arabidopsis* roots were collected from the PPRD (http://ipf.sustech.edu.cn/pub/plantrna/, accessed on 20 May 2024). (**B**) The expression patterns of *OsCASP* genes in different root cells (epidermis, cortex, endodermis, stem, root cap and metaxylem). The single-cell transcriptome data of the *OsCASP* genes in rice roots were collected from the Root Cell Atlas in Rice (RCAR) (http://www.elabcaas.cn/rcar/index.html, accessed on 20 May 2024). The heatmap was constructed by Tbtools v2.121 software (Beijing, China). The red and blue boxes indicate high and low expression levels of the *AtCASP* and *OsCASP* genes, respectively.

The analysis of the *CASP* gene expression patterns in specific root cell types of *Arabidopsis* and rice further deepens our understanding of their roles in plant root development. As illustrated in Figure 7, several *CASP* genes, including *AtCASP6* and *AtCASP_like1/13/19/31*, exhibit highly specific expression in the endodermal cells of the *Arabidopsis* roots. This preferential expression pattern suggests that these genes may play crucial roles in the development and function of the endodermis. It is worth noting that *AtCASP1/2/3/4/5* and *OsCASP1/2/3/4/5* are expressed in different root cells, but their expression levels in endodermal cells are higher than in other cells (Figure 7A). This suggests that these genes may play important roles in the development of the root endodermis in *Arabidopsis*, which is consistent with previous studies [8]. As illustrated in Figure 7B, we found that *OsCASP1* and *OsCASP_like9/11* are highly expressed in the endodermis (Figure 7B), suggesting that these genes may be involved in the development of the endodermis CS. This suggests that *OsCASP1* and *OsCASP_like9/11* may play important roles in root endodermis cell development in rice, which is consistent with previous studies of *OsCASP1*. Collectively, these results contribute to a more comprehensive picture of the regulatory mechanisms and functions of *CASP* genes in plant root development. Further studies investigating the specific functions of the individual *CASP* genes and their interactions with other regulatory factors will provide valuable insights into the mechanisms underlying plant root development and adaptation.

### 2.7. The Expression Patterns of OsCASP Genes in Different Abiotic Stresses by Transcriptome Data

**Figure 8:**
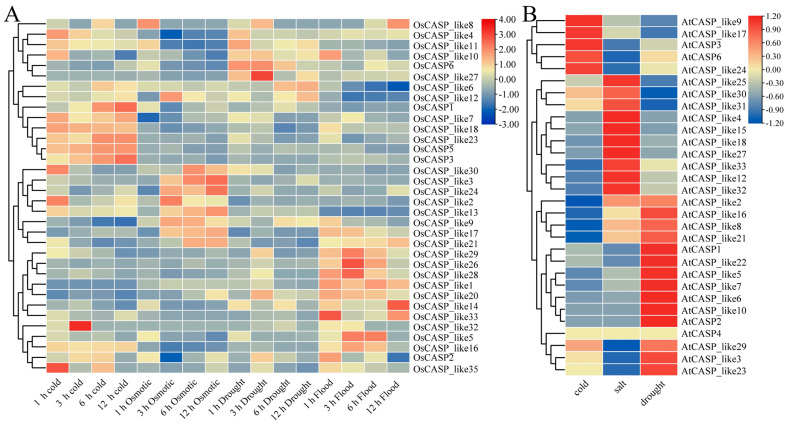
The expression patterns of *OsCASP* and *AtCASP* genes under different abiotic treatments. (**A**) The data are shown in a heatmap with the *OsCASP* gene expression in cold, osmotic, drought and flood treatments with row-scaled FPKM values. (**B**) The data are shown in a heatmap with the *AtCASP* gene expression in cold, salt and drought treatments with row-scaled FPKM values. The heatmap is constructed by Tbtools v2.121 software (Beijing, China). The red and blue boxes indicate high and low expression levels of the *OsCASP* and *AtCASP* genes, respectively.

Rice is one of the three main grains in the world; in the field environment, it is quickeasy to suffer the impact of extreme environment and reduce production. Moreover, *CASP* genes play an important role in abiotic stress, including salt, cold and Cd tolerance [23,24]. In order to screen the potential candidate *OsCASP* genes in response to abiotic stress, we mined the RNA-seq data of rice under cold, osmotic, flood and drought stresses. As shown in Figure 8A, once the rice plants were treated with cold, osmotic, drought and flood stress, sixteen, eight, ten and thirteen *OsCASP* genes were up-regulated, and seven, eleven, five and four *OsCASP* genes were down-regulated, respectively (Figure 8 and Supplemental Table S7). The expression patterns of some *OsCASP* genes showed a trend of up-regulation and then down-regulation (Figure 8 and Supplemental Table S7). The relative expression fold of *OsCASP3* was extremely up-regulated (more than 7-fold) under cold treatment compared with the control (Figure 8 and Supplemental Table S7). The expression levels of *OsCASP_like1* were down-regulated (more than 10-fold) under cold stress (Figure 8 and Supplemental Table S8). After flood treatment, the expression levels of *OsCASP_like16* and *OsCASP_like26* peaked at 3 h and then decreased. After flood and osmotic treatment, the expression levels of *OsCASP_like2*, *OsCASP_like9* and *OsCASP_like12* were the most significantly up-regulated at 3 h, while the expression levels of *OsCASP_like3*, *OsCASP_like17* and *OsCASP_like32* were at their maximums at 12 h (Figure 8 and Supplemental Table S7). Interestingly, the expression patterns of some *OsCASP* genes showed the opposite trend after being subjected to cold and drought stress (Figure 8 and Supplemental Table S7). For example, *OsCASP_like18* and *OsCASP_like23* were up-regulated and down-regulated under cold and drought stress, respectively (Figure 7 and Supplemental Table S7).

As shown in Figure 8B, we found that seven, nine and ten *AtCASP* genes were up-regulated, while eleven, five and seven *AtCASP* genes were down-regulated, respectively, under cold, salt and drought stresses (Figure 8 and Supplemental Table S7). The relative expression fold of *AtCASP_like24* was extremely up-regulated (more than 44-fold and 21.3-fold) under cold and drought treatment compared with the control, respectively (Figure 8 and Supplemental Table S7). Interestingly, 17% and 28% of the *AtCASPs* were up-regulated and down-regulated, respectively, under cold stress. Conversely, 39 percent of *OsCASPs* were raised under cold stress, and 17 percent of *OsCASPs* were down-regulated under cold stress. These results suggest that the *CASP* genes of *Arabidopsis* and rice may have different functions in response to cold stress (Figure 8 and Supplemental Table S7). Based on the above results, *OsCASP* and *AtCASP* genes play an important role in environmental stresses. These results provide valuable views for the functional characterization of the *OsCASP* and *AtCASP* genes in other plants.

### 2.8. The Expression Patterns of OsCASP Genes in Ion Defects by RNA-Seq Data

**Figure 9:**
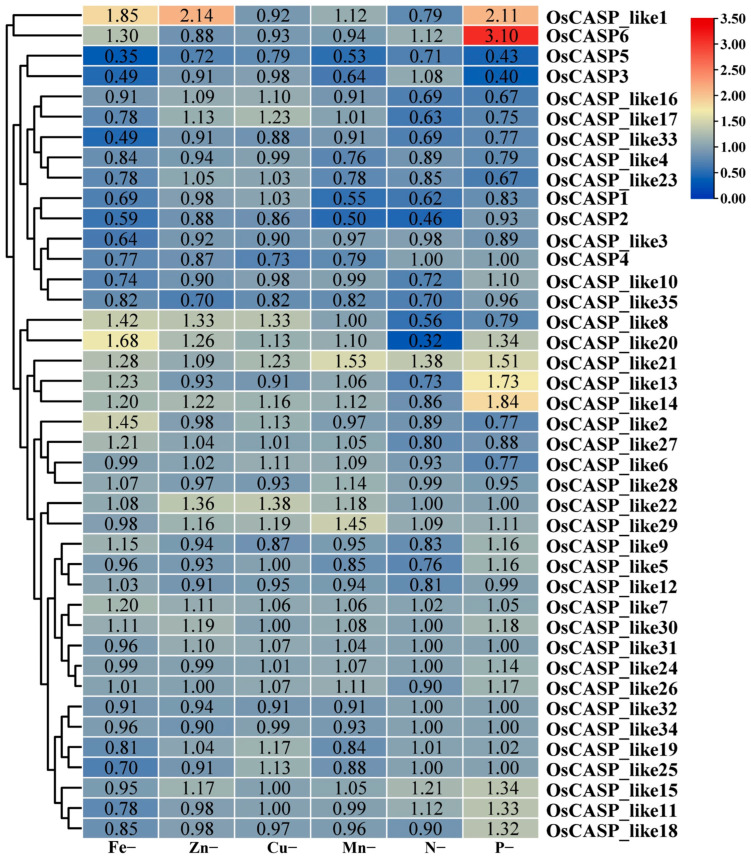
The expression patterns of *OsCASP* genes under iron deficiency, zinc deficiency, copper deficiency, manganese deficiency, nitrogen deficiency and phosphorus deficiency treatment by RNA-seq. The heatmap was constructed by Tbtools v2.121 software(Beijing, China). The red and blue boxes indicate high and low expression levels of the *OsCASP* genes, respectively.

Previous studies have shown that *CASP* genes play an important role in ion absorption [4,5,24]. In order to further explore the possible effects of *OsCASP* genes in the process of ion defects, we analyzed the expression patterns of the *OsCASP* genes using root transcriptome data from rice under iron deficiency, zinc deficiency, copper deficiency, manganese deficiency, nitrogen deficiency and phosphorus deficiency. As shown in Figure 9, some *OsCASP* genes exhibited the same expression patterns under iron deficiency, zinc deficiency, copper deficiency, manganese deficiency, nitrogen deficiency and phosphorus deficiency. For instance, the expression levels of the *OsCASP_like8*, *OsCASP_like14*, *OsCASP_like20* and *OsCASP_like21* genes were up-regulated under iron deficiency, zinc deficiency, copper deficiency and manganese deficiency (Figure 8 and Supplemental Table S8). Moreover, the expression levels of the *OsCASP6*, *OsCASP_like11*, *OsCASP_like15* and *OsCASP_like21* genes were up-regulated under nitrogen deficiency and phosphorus deficiency (Figure 8 and Supplemental Table S8). Interestingly, some *OsCASP* genes exhibited the opposite expression patterns under iron deficiency, zinc deficiency, copper deficiency, manganese deficiency, nitrogen deficiency and phosphorus deficiency. For example, *OsCASP6* and *OsCASP_like11* showed an up-regulation trend under nitrogen deficiency and phosphorus deficiency but a down-regulation trend under iron deficiency, zinc deficiency, copper deficiency and manganese deficiency (Figure 8 and Supplemental Table S8), whereas the expression levels of *OsCASP_like6*, *OsCASP_like17*, *OsCASP_like23* and *OsCASP_like27* were down-regulated under nitrogen deficiency and phosphorus deficiency but up-regulated under iron deficiency, zinc deficiency, copper deficiency and manganese deficiency (Figure 9 and Supplemental Table S8). These results suggest that these *OsCASP* genes may be involved in ion defect processes in opposite patterns. Previous studies have found that nitrogen and phosphorus can coordinate plant growth and development [25]. Our study found that *OsCASP_like4*, *OsCASP_like16*, *OsCASP_like17* and *OsCASP_like33* showed a down-regulation trend under nitrogen deficiency and phosphorus deficiency. It implies that these genes may be coordinated to regulate the processes of nitrogen and phosphorus deficiency.

### 2.9. Expression Patterns of OsCASP Genes in Different Ion Defects by RT-qPCR

**Figure 10:**
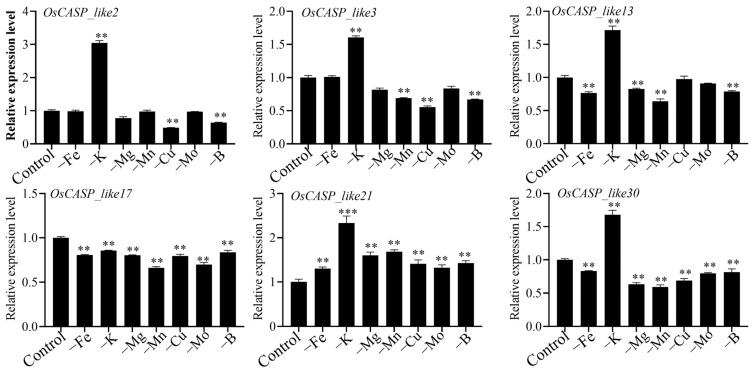
The relative expressions of the *OsCASP* genes were detected by RT-qPCR under iron deficiency, potassium deficiency, magnesium deficiency, manganese deficiency, copper deficiency, molybdenum deficiency and boron deficiency treatment. The −Fe, −K, −Mg, −Mn, −Cu, −Mo and −B represent iron deficiency, potassium deficiency, magnesium deficiency, manganese deficiency, copper deficiency, molybdenum deficiency and boron deficiency, respectively. The rice seedlings were cultured with Kimura B solution for 7 days, then transferred into normal solution, −Fe (0 µM Fe (II)-EDTA), −K (0 mM K_2_SO_4_), −Mg (0 mM MgSO_4_·7H_2_O), −Mn (0 µM MnCl_2_·4H_2_O), −Cu (0 µM CuSO_4_·5H_2_O), −Mo (0 µM (NH_4_)_6_Mo_7_O_24_·4H_2_O), or −B (0 µM H_3_BO_3_) for 3 days, respectively. Samples were obtained at 3 days post-treatment. All dates are the means ± SD of three biological replicates. The significance analysis was calculated by Prism 9 software (using Student’s *t*-test, ** *p* < 0.01, *** *p* < 0.001). *OsActin* was used as the internal reference for the RT-qPCR analysis.

In order to further explore the processes of the *OsCASP* genes involved in the ion defects, we randomly selected six *OsCASP* genes and tested their expression patterns in ion deficiency by RT-qPCR, for the following reasons: firstly, the *OsCASP* genes were expressed in the root; secondly, the *OsCASP* genes were expressed under the conditions of ion deficiency. As shown in Figure 10, the expression levels of the *OsCASP_like2*, *OsCASP_like3*, *OsCASP_like13*, *OsCASP_like21* and *OsCASP_like30* genes were up-regulated under potassium deficiency, whereas the expression levels of *OsCASP_like2*, *OsCASP_like3*, *OsCASP_like17* and *OsCASP_like30* were down-regulated under copper deficiency and boron deficiency (Figure 10). Interestingly, compared to the control group, the expression level of *OsCASP_like21* was significantly increased in the case of ionic defects (Figure 10). Moreover, except for the potassium deficiency, the expression level of *OsCASP_like30* was up-regulated, while the rest of the ion defects were decreased (Figure 10). It is worth noting that some *OsCASP* genes exhibited the opposite expression pattern under ion defect. For example, *OsCASP_like17* showed a down-regulation trend under iron deficiency, potassium deficiency, magnesium deficiency, manganese deficiency, copper deficiency, molybdenum deficiency and boron deficiency, whereas the expression level of *OsCASP_like21* was up-regulated under ion defects (Figure 10). On the other hand, some *OsCASP* genes exhibited the same expression pattern under ion defects, such as the expression levels of the *OsCASP_like3*, *OsCASP_like21* and *OsCASP_like30* genes being down-regulated under manganese deficiency, copper deficiency and boron deficiency.

## 3. Discussion

The *CASP* genes plays an important role in the endodermal CS formation of roots and are ubiquitously present across various plant species [4,5,8,20]. Notably, the number of *CASP* genes exhibits significant variation among different plant species. For instance, the counts of *CASP* genes in *Arabidopsis*, *G. arboretum* and *P. cablin* are 39, 48 and 156, respectively [8,19,20]. The number of *CASP* genes in rice was more than that of *Arabidopsis* and *litchi* and less than that of *G. arboreum* and *P. cablin* (Supplemental Table S1). The likely reason is that *G. arboreum* has a more complex genome than rice and *Arabidopsis* [8]. *P. cablin* has the most *CASP* genes because it is a tetraploid plant [20]. In the process of evolution, TD and WGD events not only provide opportunities for gene evolution and functional innovation, but also promote the adaptive evolution of species [26,27]. About 70 million years ago, the rice genome underwent a pattern of replication dominated by tandem replication [28,29]. Through analysis, we identified three and TD and six and six WGD gene pairs in rice and *Arabidopsis*, respectively (Figure 4). This suggests that TDs and WGDs could have contributed to the expansion of the *AtCASP* and *OsCASP* gene families in rice and *Arabidopsis*, but the latter play a major role. Furthermore, homologous *CASP* genes across species may retain similar biological functions during evolution. As shown in Figure 4E, six homologous *CASP* gene pairs were identified in rice and *Arabidopsis*. On the other hand, we found that OsCASP_like6, OsCASP_like13 and OsCASP_like18 shared close relationships with AtCASP_like16, AtCASP_like17 and AtCASP_like29 (Figure 2), indicating potential functional similarities among these genes. This hypothesis is supported by previous studies, which have shown that OsCASP1 shares close relationships with AtCASP1 and AtCASP3, both of which regulate the formation of the endodermal CS [4].

Gene structure is a pivotal determinant of gene function [30]. Within the CASP gene family, the genes belonging to the same subgroup exhibit similar structures and conserve common motifs, whereas the genes from distinct subfamilies display marked structural differences. Interestingly, we observed variations in the conserved domains of the *CASP* gene family across rice and *Arabidopsis*. Specifically, *Arabidopsis* possesses the MARVEL domain, which is absent in rice (Figure 3B). This finding is consistent with previous research conducted on *P. cablin*, banana and litchi, further validating our observations [20]. These data suggest that *CASP* genes are both functionally conserved and functionally differentiated in different species.

As shown in Figure 5, the promoter regions of most *CASP* genes contain hormone response, development response and stress response elements, and we speculated that the CASP gene family may be involved in the regulation of growth and development and abiotic and biotic stresses. The transcriptome data further confirmed our speculations, and the transcription levels of some *OsCASP* genes were significantly up-regulated and down-regulated by drought, cold and osmotic stress (Figure 8). Similarly, previous studies have found that the expression of *CASPs* can respond to salt, osmotic and cold treatments [23,24]. Moreover, *AtCASPL4C1* and *SbCASP-LP1C1* play a key role in cold and salt tolerance [24]. Previous studies have shown that the overexpression of *OsCASP1* improves calcium tolerance in rice, while the knockout of the *OsCASP1* gene decreases tolerance to potassium and magnesium deficiency. We found that the *OsCASP_like17* and *OsCASP_like21* genes exhibited opposite expression patterns under iron deficiency, potassium deficiency, magnesium deficiency, manganese deficiency, copper deficiency, molybdenum deficiency and boron deficiency, implying that *OsCASP_like17* and *OsCASP_like21* may antagonist regulation these ion defects processes (Figure 10). In addition, the *OsCASP_like3*, *OsCASP_like21* and *OsCASP_like30* genes exhibited the same expression pattern under manganese deficiency, copper deficiency and boron deficiency, indicating these genes may play a similar function in these ion defect processes (Figure 10). Notably, we found a large number of light-responsive elements in the promoter regions of the *OsCASP* and *AtCASP* genes. These results may imply that *CASP* genes not only play an important role in the development of the CS and stress response, but also play an important role in the photopogenesis process, which needs to be further studied.

The transcription factors AtMYB36 and OsMYB36a/b/c positively regulate the expression of CS-related genes (AtCASP1/3, AtESB1, AtPER64 and OsCASP1) [5,13]. In *Arabidopsis* and rice, 67.57% and 85.37% of *CASP* gene promoter regions contain the sequence of the MYB binding motif, respectively (Figure 5 and Supplemental Table S2). In this study, we found that *OsCASP_like11*, *OsCASP_like9*, *AtCASP_like1* and *AtCASP_like31* were specifically highly expressed in endodermis cells (Figure 7). Moreover, the MYB binding motif “CAACC”, a sequence known to be CS-related, was present in the promoter regions of *OsCASP_like11*, *OsCASP_like9*, *AtCASP_like1* and *AtCASP_like31* as previously described [5,13]. Therefore, we speculated that *OsCASP_like11*, *OsCASP_like9*, *AtCASP_like1* and *AtCASP_like31* might be candidate genes involved in the formation of endodermis CSs. Previous studies have shown that CSs are found in the stems and leaves of ferns [31]. A recent study for the first time identified a new apoplastic barrier cell wall structure composed of lignin, neck strip, that regulates the formation of cucumber peel wax powder in non-root cells [32]. Our study found that some *AtCASP* and *OsCASP* genes are highly expressed in leaves, suggesting that these genes may be involved in the development of the leaves’ CSs, which is a bold guess and worthy of further exploration.

## 4. Methods

### 4.1. Identification, Chromosomal Location and Phylogenetic Analysis of AtCASPs and OsCASPs

The genome sequence and genome annotation (GFF) files of *A. thaliana* and *O. sativa* were downloaded from the phytozome v13 database (https://Phytozome-next.jgi.doe.gov, accessed on 9 May 2024) [33]. The longest transcripts of *Arabidopsis* and rice were obtained by Tbtools v2.121 software (Beijing, China) and translated into protein sequences [22]. The hidden Markov model of the DUF588 domain (PF04535) was obtained from the PFAM database (http://pfam.sanger.ac.uk/, accessed on 9 May 2024) [34], and HMMER 3.0 software was used to search the *CASP* genes in the *A. thaliana* and *O. sativa* protein database. Afterwards, these CASP sequences were submitted to NCBI-CDD (https://www.ncbi.nlm.nih.gov/Structure/bwrpsb/bwrpsb.cgi, accessed on 9 May 2024) to confirm the presence of the conserved DUF588 and MARVEL domains. The chromosome positions of the *AtCASP* and *OsCASP* genes were obtained from the GFF files, and Tbtools v2.121 software was used for visualization [22]. The AtCASP and OsCASP sequences were used to perform a phylogenetic analysis by MEGA 7.0 using the neighbor joining (N-J) method [35].

### 4.2. Gene Structure and Conserved Motifs Analysis of AtCASPs and OsCASPs

The gene structures of the *AtCASP* and *OsCASP* genes were obtained from the GFF files, and the conserved motifs of the AtCASPs and OsCASPs were elucidated by the MEME [36]. The picture of the gene structures and protein conserved motifs was visualized using Tbtools v2.121 (Beijing, China) [22].

### 4.3. Duplication Events, Ka/Ks and Synteny Analysis of AtCASPs and OsCASPs

The MCScan X (Beijing, China) was used to analyze segmental duplications and tandem duplications [37]. The Ka/Ks ratios and orthologous gene pairs were detected by Tbtools v2.121 (Beijing, China) [22].

### 4.4. Cis-Acting Element Analysis of AtCASPs and OsCASPs

The PlantCARE database (http://bioinformatics.psb.ugent.be/webtools/plantcare/html/, accessed on 15 May 2024) was used to analyze the cis-acting elements in the *AtCASP* and *OsCASP* promoter regions (2 kb upstream of the initiation codon ATG) [38].

### 4.5. Expression Patterns of AtCASP and OsCASP Genes with RNA-Seq

The gene expression patterns of the different tissues (rice: root, stem, panicle before flowering, panicle after flowering and flag leaf; *Arabidopsis*: root: cotyledon, leaf blade, leaf midrib, leaf petiole and inflorescence) were downloaded from the Plant Public RNA-seq Database (PPRD, http://ipf.sustech.edu.cn/pub/plantrna/, accessed on 18 May 2024) [39]. The rice breed was *Oryza sativa* ssp. *Japonica* cv (Nipponbare); the seeds were germinated in a growth chamber at 28 °C under a 16 h light/8 h dark regime. Seven days after germination, the roots were collected. In the meantime, plants were grown in paddy fields, where the flag leaf, panicle after flowering, panicle after flowering and stem were collected. he *Arabidopsis thaliana* breed: Col-0, to promote germination, the seeds were stratified in 1/2 vermiculite/soil at 4 °C for five days. The plants were grown in a climate chamber under a 16 h light/8 h dark cycle at 22 °C and 50% relative humidity. Then, the gene expression levels were calculated using FPKM. The single-cell transcriptome data of rice were downloaded from the Root Cell Atlas in Rice (RCAR) (http://www.elabcaas.cn/rcar/index.html, accessed on 20 May 2024) [40]. For the gene expression patterns of different abiotic stresses (cold, osmotic, drought and flood), the rice breed was *Oryza sativa* ssp. *Japonica* cv (Nipponbare); the seeds were germinated in a growth chamber at 28 °C under a 16 h light/8 h dark regime. Seven days after germination, they were transferred to a culture nutrient solution for cold, osmotic, drought and flood treatment. Samples were obtained at 1 h, 3 h, 6 h and 12 h for the cold, osmotic, drought and flood treatments, respectively.

### 4.6. Plant Materials, Growth Conditions and Treatments

Seedlings of the rice cultivar Zhonghua 11 (*O. sativa*, ZH11) were cultivated in a greenhouse under the conditions of 12/12 h light/dark (200 µmol m^−2^ s^−1^), 28 °C and 70% humidity. The seeds after two days of germination in water at 37 °C were grown in black boxes as supporting materials in a modified Kimura B solution [25], and the Kimura B solution was replaced every two days.

For the iron deficiency, magnesium deficiency, manganese deficiency, copper deficiency, molybdenum deficiency and boron deficiency treatments, the rice seedlings were cultured with Kimura B solution for seven days, then transferred into normal solution, iron deficiency (0 µM Fe (II)-EDTA), potassium deficiency (0 mM K_2_SO_4_), magnesium deficiency (0 mM MgSO_4_·7H_2_O), manganese deficiency (0 µM MnCl_2_·4H_2_O), copper deficiency (0 µM CuSO_4_·5H_2_O), molybdenum deficiency (0 µM (NH_4_)_6_Mo_7_O_24_·4H_2_O), or boron deficiency (0 µM H_3_BO_3_) for three days, respectively. Samples were obtained at three days post-treatment.

### 4.7. RNA Extraction and RT-qPCR Analysis

In our study, primer 5.0 software was used for the design of an *OsCASP* gene-specific primer, shown in Supplemental Table S9. The rice root total RNA was extracted by KKFast Plant RNApure Kit (ZOMANBIO, Beijing, China). The cDNA was a reverse transcription synthesis by PrimerScript^TM^ IV 1st strand cDNA Synthesis Mix (TaKaRa, Nojihigashi, Japan). The RT-qPCR reaction system and program was conducted according to the protocols described by Duan et al. [21].

## 5. Conclusions

In this study, we executed a comprehensive and systemic analysis of the *CASP* genes in rice and *Arabidopsis*, and 39 *AtCASP* and 41 *OsCASP* genes were confirmed. Phylogenetic results showed that the OsCASPs and AtCASPs were clustered into six subgroups, and WGD and TD events were a major driving force for CASP evolution. Moreover, we also analyzed the expression patterns of the *OsCASP* and *AtCASP* genes. These results provide an important theoretical basis for further exploring the biological functions of the *OsCASP* and *AtCASP* families of genes and their role in Casparian strip development and abiotic stresses.

## Data Availability

Data are contained within the article and Supplementary Materials.

## Supplementary Materials

The following supporting information can be downloaded at https://www.mdpi.com/article/10.3390/ijms25189858/s1.
