# Supplementary material for: Comparative Analysis of Casparian Strip Membrane Domain Protein Family in Oryza sativa (L.) and Arabidopsis thaliana (L.)
Source: Int J Mol Sci. 2024 Sep 12;25(18):9858. doi: 10.3390/ijms25189858 (PMC11431883; doi:10.3390/ijms25189858)
Supplement: Supplementary file 1 [file ijms-25-09858-s001.zip › Supplemental figure.pdf]

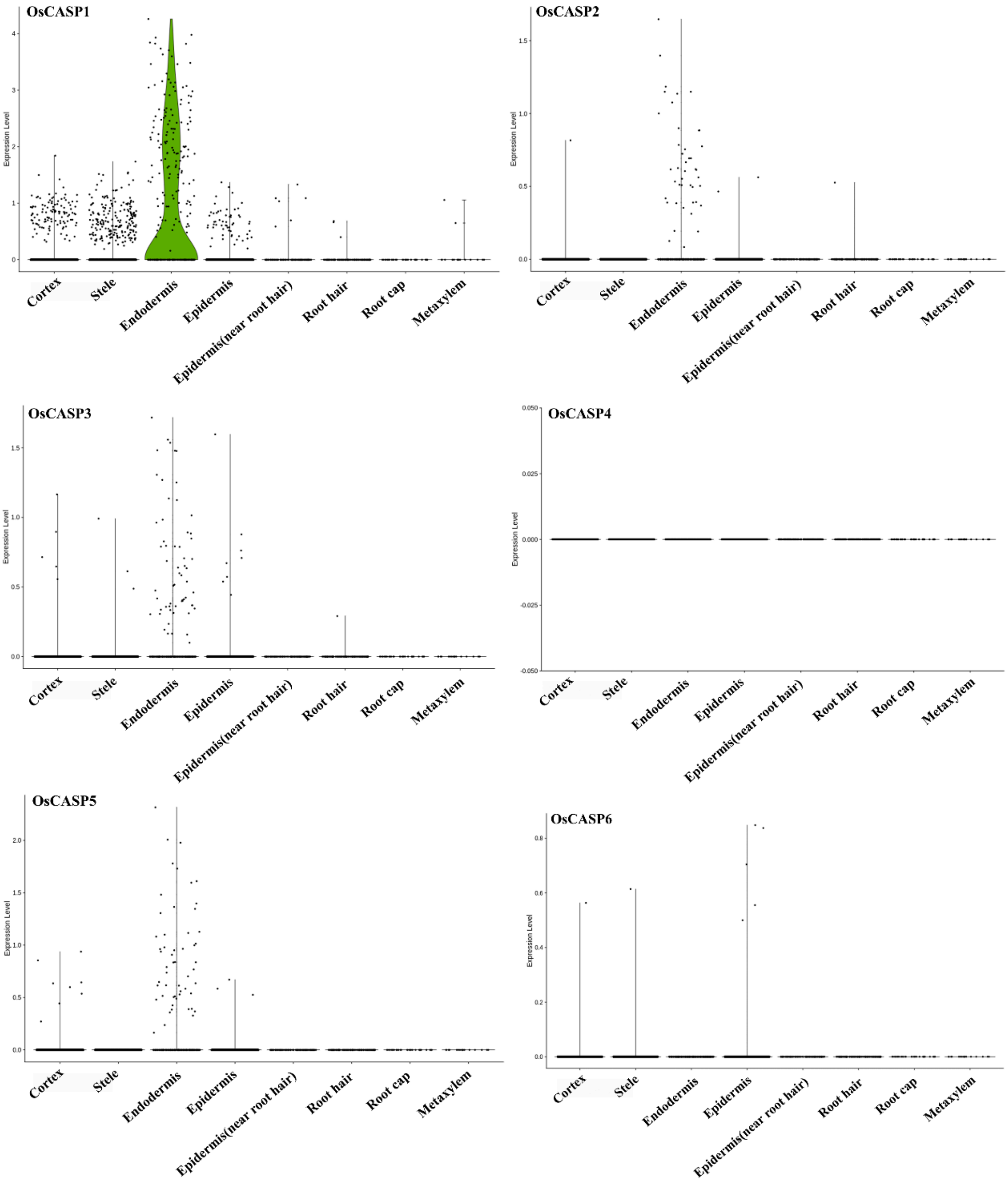
OsCASP1
Expression Level
Cortex
Stele
Endodermis
Epidermis
Epidermis(near root hair)
Root hair
Root cap
Metaxylem
OsCASP2
Expression Level
Cortex
Stele
Endodermis
Epidermis
Epidermis(near root hair)
Root hair
Root cap
Metaxylem
OsCASP3
Expression Level
Cortex
Stele
Endodermis
Epidermis
Epidermis(near root hair)
Root hair
Root cap
Metaxylem
OsCASP4
Expression Level
Cortex
Stele
Endodermis
Epidermis
Epidermis(near root hair)
Root hair
Root cap
Metaxylem
OsCASP5
Expression Level
Cortex
Stele
Endodermis
Epidermis
Epidermis(near root hair)
Root hair
Root cap
Metaxylem
OsCASP6
Expression Level
Cortex
Stele
Endodermis
Epidermis
Epidermis(near root hair)
Root hair
Root cap
Metaxylem

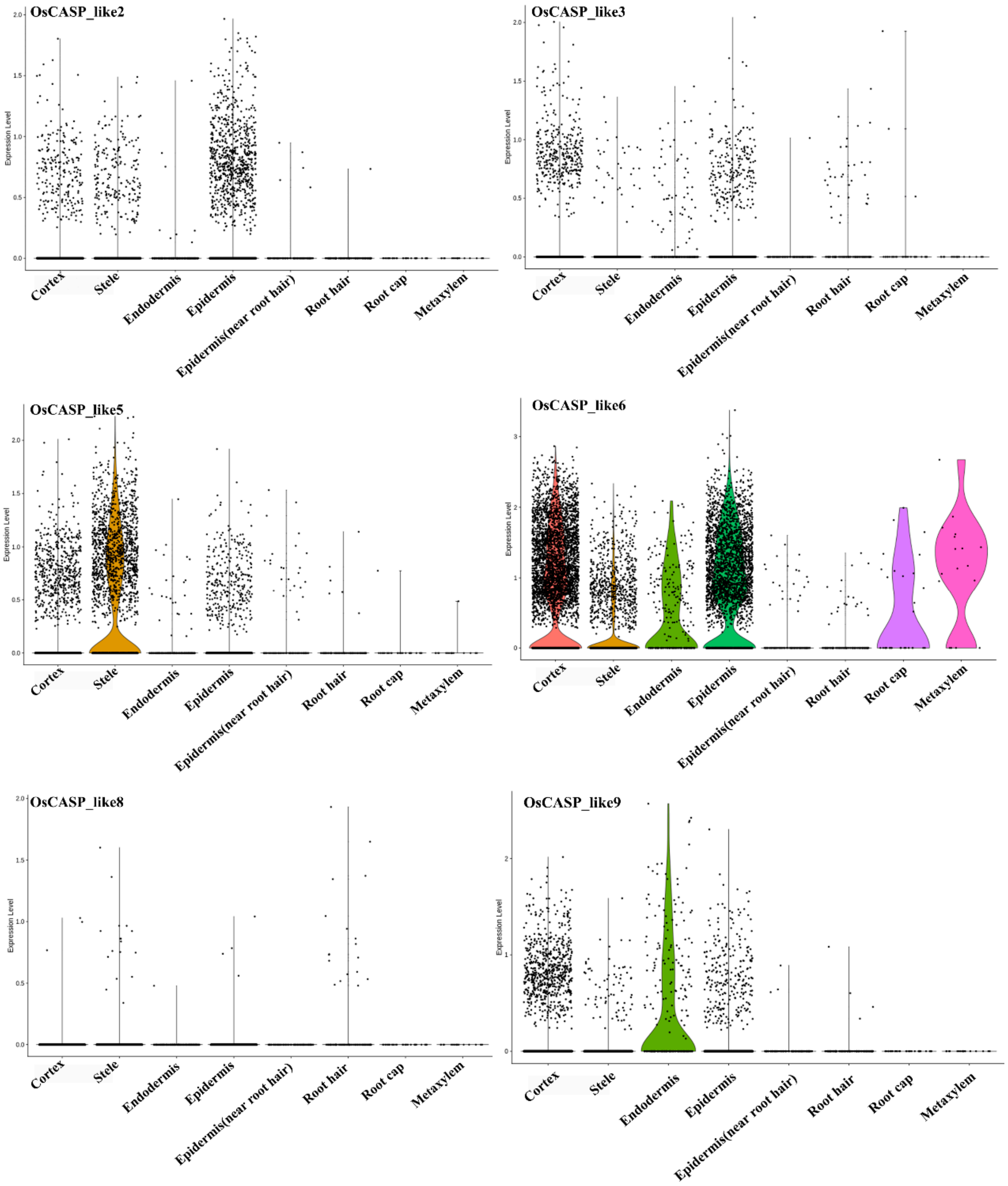
OsCASP_like2
OsCASP_like3
OsCASP_like5
OsCASP_like6
OsCASP_like8
OsCASP_like9
Expression Level
Cortex
Stele
Endodermis
Epidermis
Epidermis(near root hair)
Root hair
Root cap
Metaxylem

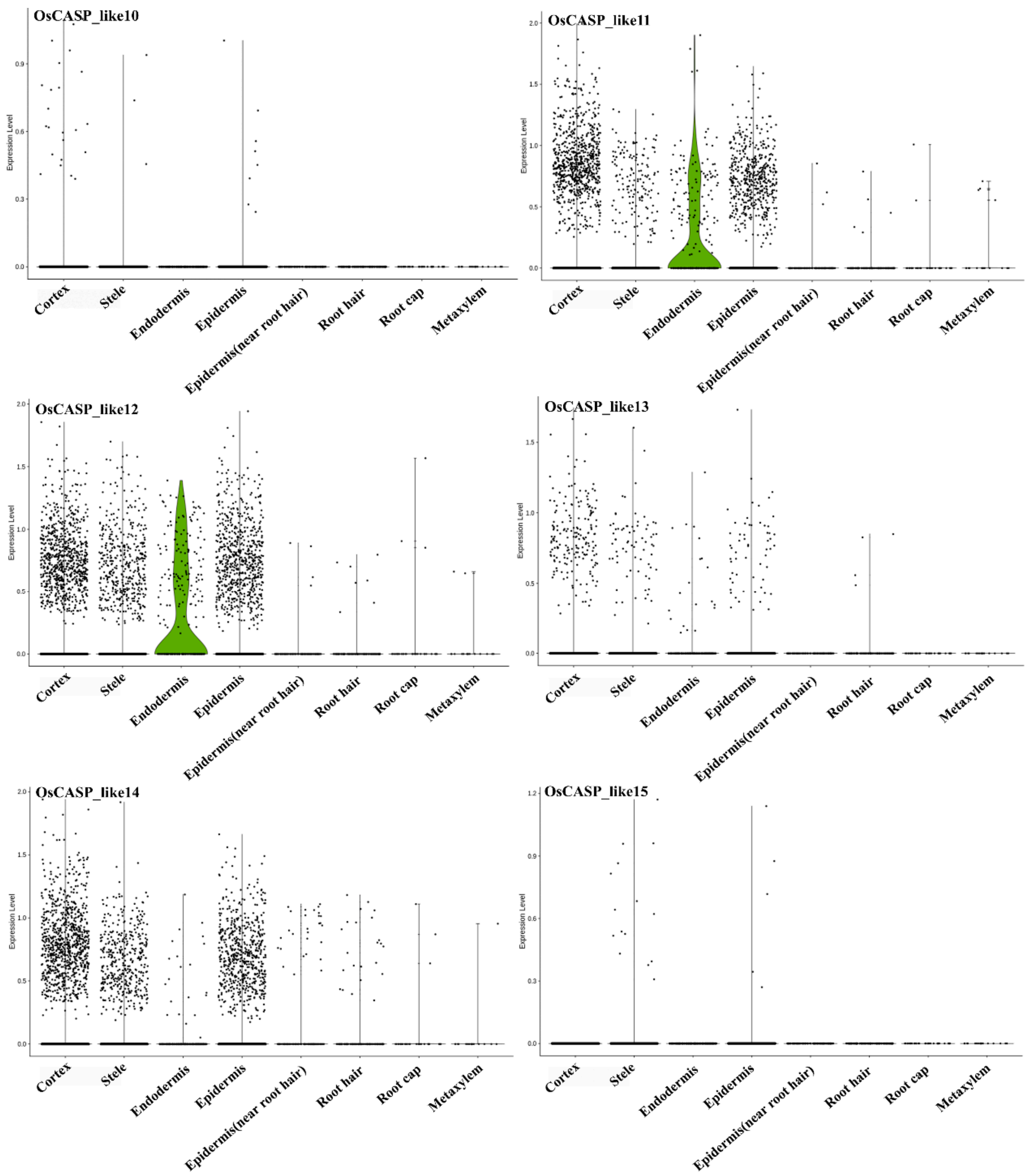
OsCASP_like10
Expression Level
Cortex
Stele
Endodermis
Epidermis
Epidermis(near root hair)
Root hair
Root cap
Metaxylem
OsCASP_like11
Expression Level
Cortex
Stele
Endodermis
Epidermis
Epidermis(near root hair)
Root hair
Root cap
Metaxylem
OsCASP_like12
Expression Level
Cortex
Stele
Endodermis
Epidermis
Epidermis(near root hair)
Root hair
Root cap
Metaxylem
OsCASP_like13
Expression Level
Cortex
Stele
Endodermis
Epidermis
Epidermis(near root hair)
Root hair
Root cap
Metaxylem
OsCASP_like14
Expression Level
Cortex
Stele
Endodermis
Epidermis
Epidermis(near root hair)
Root hair
Root cap
Metaxylem
OsCASP_like15
Expression Level
Cortex
Stele
Endodermis
Epidermis
Epidermis(near root hair)
Root hair
Root cap
Metaxylem

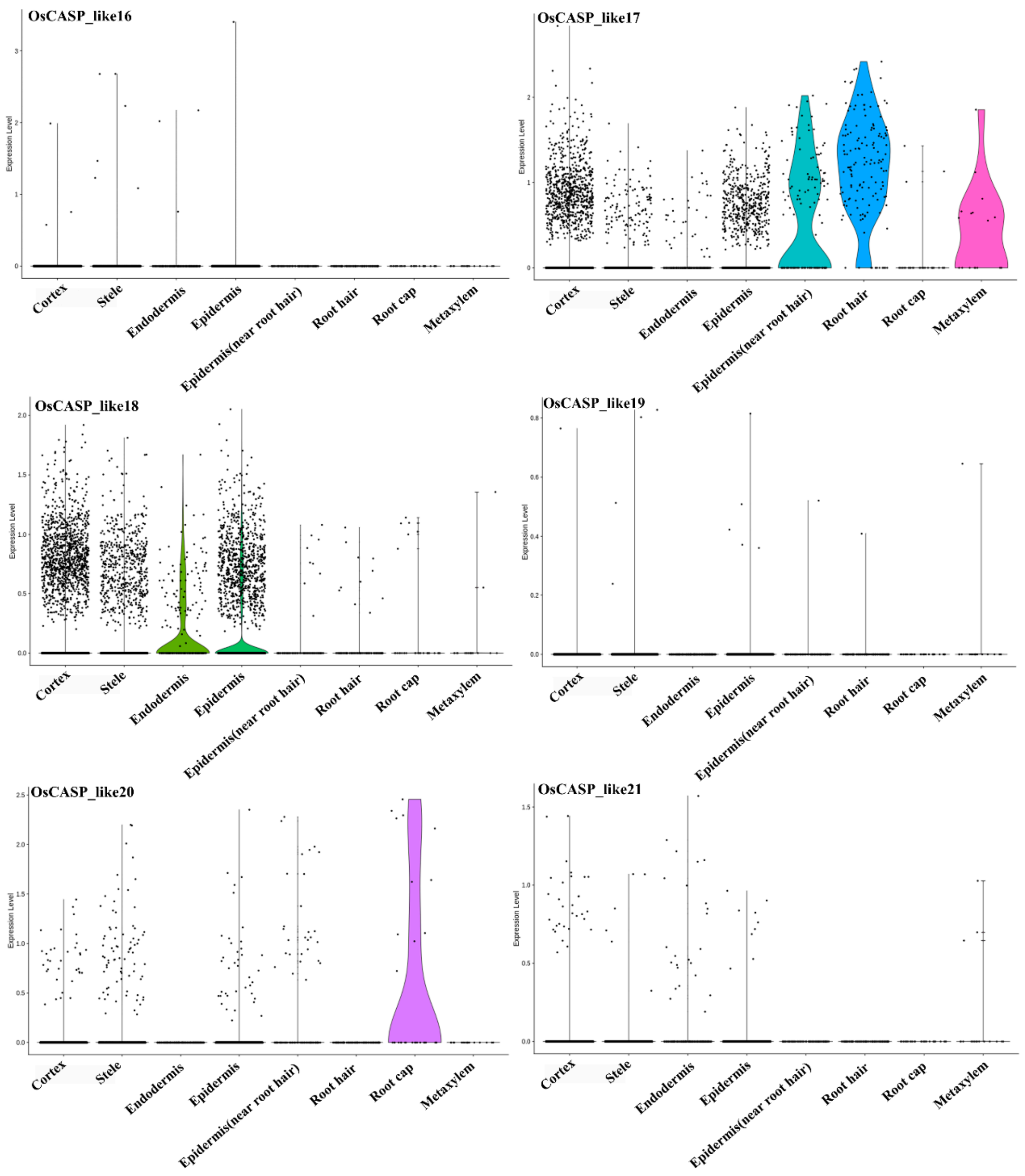
OsCASP_like16
Expression Level
Cortex
Stele
Endodermis
Epidermis
Epidermis(near root hair)
Root hair
Root cap
Metaxylem
OsCASP_like17
Expression Level
Cortex
Stele
Endodermis
Epidermis
Epidermis(near root hair)
Root hair
Root cap
Metaxylem
OsCASP_like18
Expression Level
Cortex
Stele
Endodermis
Epidermis
Epidermis(near root hair)
Root hair
Root cap
Metaxylem
OsCASP_like19
Expression Level
Cortex
Stele
Endodermis
Epidermis
Epidermis(near root hair)
Root hair
Root cap
Metaxylem
OsCASP_like20
Expression Level
Cortex
Stele
Endodermis
Epidermis
Epidermis(near root hair)
Root hair
Root cap
Metaxylem
OsCASP_like21
Expression Level
Cortex
Stele
Endodermis
Epidermis
Epidermis(near root hair)
Root hair
Root cap
Metaxylem

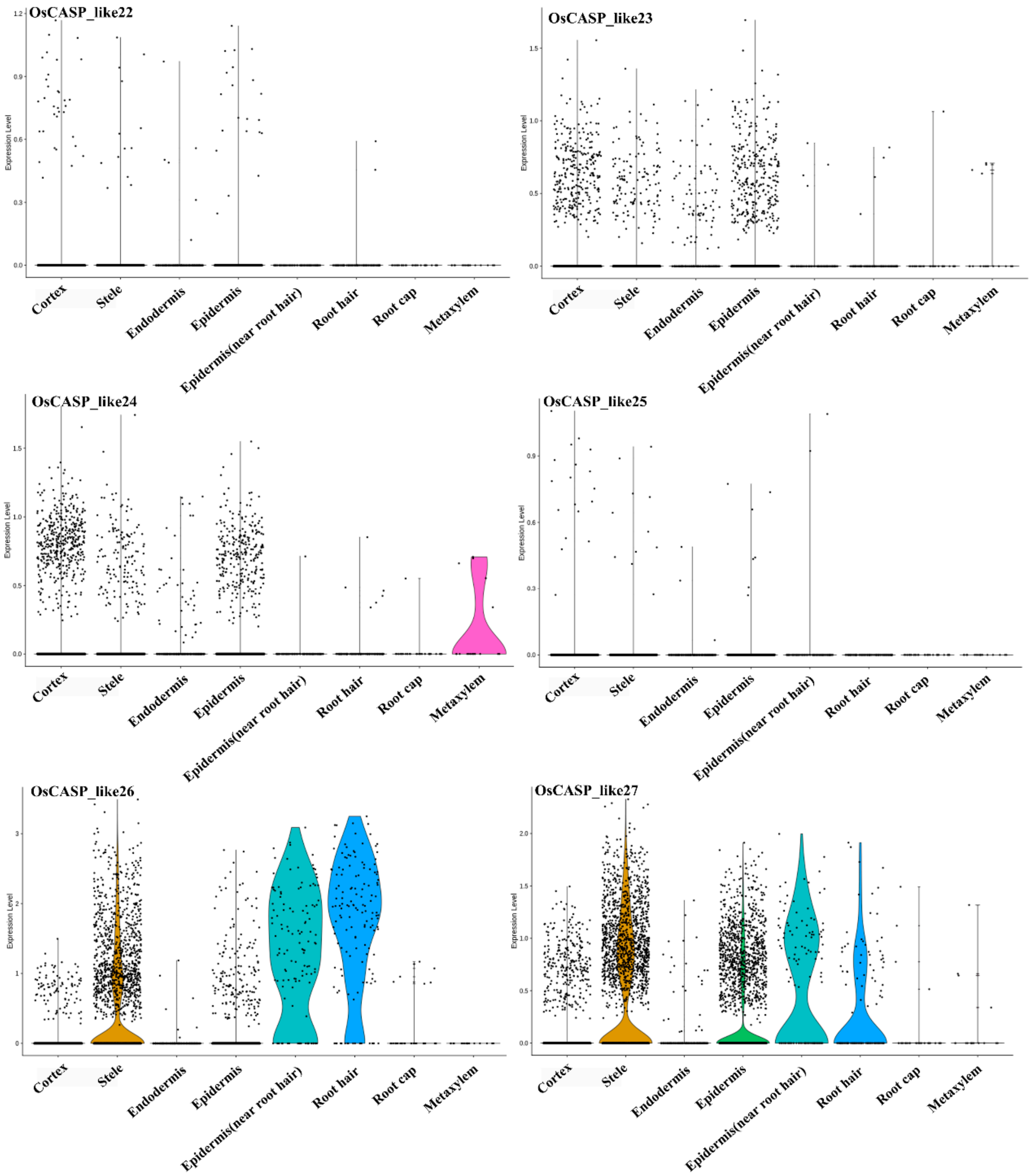
OsCASP_like22
OsCASP_like23
OsCASP_like24
OsCASP_like25
OsCASP_like26
OsCASP_like27
Expression Level
Cortex
Stele
Endodermis
Epidermis
Epidermis(near root hair)
Root hair
Root cap
Metaxylem

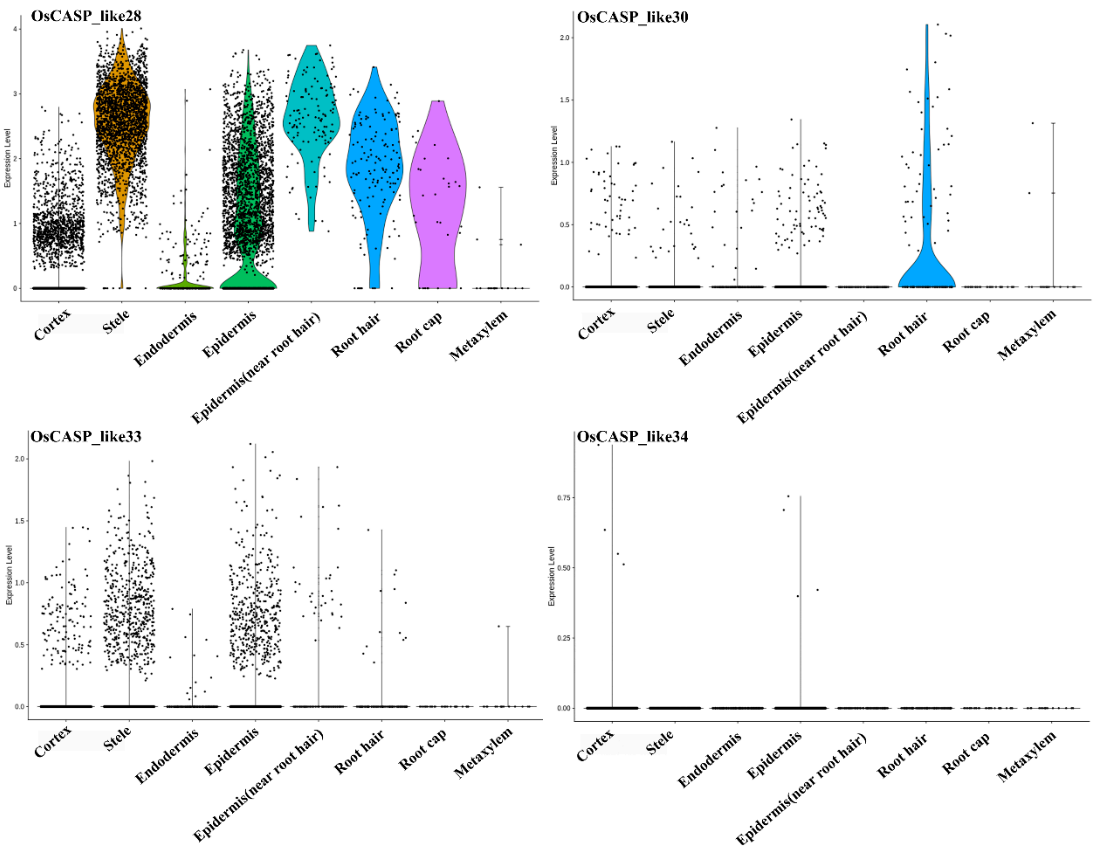

Supplemental Figure S1. Expression pattern of *CASPs* genes in rice toot cells

Single cell transcriptome data of CASPs in rice roots. The expression data were collected from the Root Cell Atlas in Rice (RCAR) (http://www.elabcaas.cn/rcar/index.html).

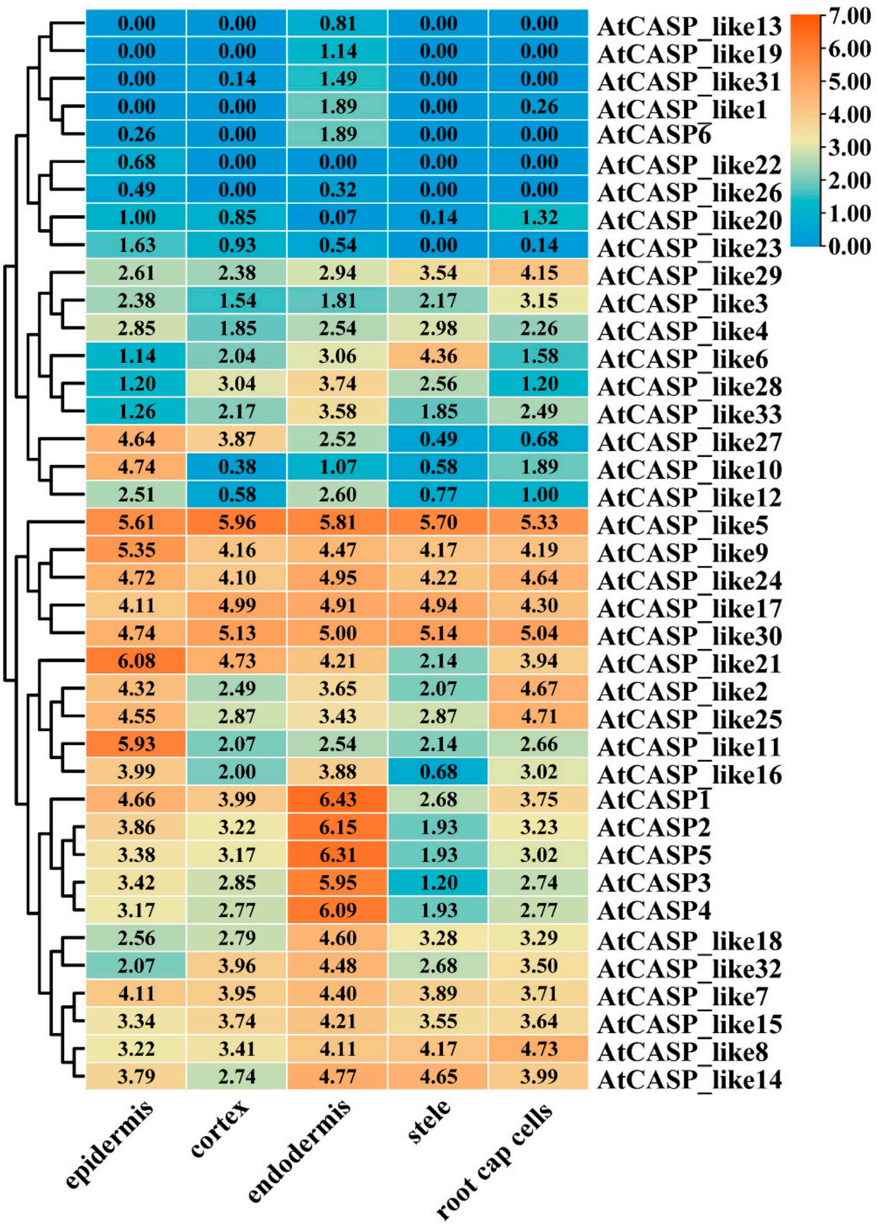

Supplemental Figure S2. Expression pattern of *CASPs* genes in *Arabidopsis* root cells.

Single cell transcriptome data of CASPs in *Arabidopsis* roots. The expression data were collected from the PPRD (http://ipf.sustech.edu.cn/pub/plantrna/).
